# Identification and Expression Analysis of Hormone Biosynthetic and Metabolism Genes in the 2OGD Family for Identifying Genes That May Be Involved in Tomato Fruit Ripening

**DOI:** 10.3390/ijms21155344

**Published:** 2020-07-28

**Authors:** Qiangqiang Ding, Feng Wang, Juan Xue, Xinxin Yang, Junmiao Fan, Hong Chen, Yi Li, Han Wu

**Affiliations:** 1 State Key Laboratory of Crop Genetics and Germplasm Enhancement, College of Horticulture, Nanjing Agricultural University, Nanjing 210095, China; 2016204015@njau.edu.cn (Q.D.); wangf@njau.edu.cn (F.W.); 2017104059@njau.edu.cn (J.X.); 2018104055@njau.edu.cn (X.Y.); 2018204022@njau.edu.cn (J.F.); 2 Jiangsu Key Laboratory for the Research and Utilization of Plant Resources, Institute of Botany, Jiangsu Province and Chinese Academy of Sciences, Nanjing 210014, China; ch198472@163.com; 3 Department of Plant Science and Landscape Architecture, University of Connecticut, Storrs, CT 06269, USA; yi.li@uconn.edu

**Keywords:** genome-wide identification, expression analysis, 2OGD family, hormone biosynthetic and metabolism genes, tomato fruit ripening

## Abstract

Phytohormones play important roles in modulating tomato fruit development and ripening. The 2-oxoglutarate-dependent dioxygenase (2OGD) superfamily containing several subfamilies involved in hormone biosynthesis and metabolism. In this study, we aimed to identify hormone biosynthesis and metabolism-related to 2OGD proteins in tomato and explored their roles in fruit development and ripening. We identified nine 2OGD protein subfamilies involved in hormone biosynthesis and metabolism, including the gibberellin (GA) biosynthetic protein families GA20ox and GA3ox, GA degradation protein families C19-GA2ox and C20-GA2ox, ethylene biosynthetic protein family ACO, auxin degradation protein family DAO, jasmonate hydroxylation protein family JOX, salicylic acid degradation protein family DMR6, and strigolactone biosynthetic protein family LBO. These genes were differentially expressed in different tomato organs. The GA degradation gene *SlGA2ox2*, and the auxin degradation gene *SlDAO1*, showed significantly increased expression from the mature-green to the breaker stage during tomato fruit ripening, accompanied by decreased endogenous GA and auxin, indicating that *SlGA2ox2* and *SlDAO1* were responsible for the reduced GA and auxin concentrations. Additionally, exogenous gibberellin 3 (GA_3_) and indole-3-acetic acid (IAA) treatment of mature-green fruits delayed fruit ripening and increased the expression of *SlGA2ox2* and *SlDAO1*, respectively. Therefore, *SlGA2ox2* and *SlDAO1* are implicated in the degradation of GAs and auxin during tomato fruit ripening.

## 1. Introduction

Tomato is used as a model to study climacteric fruit ripening, which is mediated by the hormone ethylene. Other hormones are also involved in tomato ripening. For example, exogenous auxin treatment, or increasing the endogenous auxin level by silencing the expression of the auxin-degradation gene *SlGH3.2*, delayed tomato fruit ripening [1,2,3]. Indeed, exogenous application of gibberellins (GAs) delayed fruit ripening, while decreasing the endogenous levels of GAs via overexpression of the GA-catabolic gene *SlGA2ox1* accelerated fruit ripening [4]. Therefore, changes in hormone concentrations play important roles in tomato fruit ripening, and identification and functional analysis of hormone biosynthetic and metabolism genes are prerequisites for understanding their roles in tomato fruit ripening.

The 2-oxoglutarate-dependent dioxygenase (2OGD) superfamily is the largest enzyme family and facilitates numerous oxidative reactions, including hydroxylation, halogenation, desaturation, epimerization, etc. [5]. The 2OGD superfamily contains many proteins involved in hormone biosynthesis and metabolism. To date, nine hormone biosynthesis and metabolism-related protein families have been identified in the 2OGD family, including GA biosynthetic protein families GA20-oxidase (GA20ox) and GA3-oxidase (GA3ox), GA degradation protein families GA2-oxidases (C19-GA2ox and C20-GA2ox), auxin degradation protein family Dioxygenase for Auxin Oxidation (DAO), ethylene biosynthetic protein family 1-aminocyclopropane-1-carboxylic acid oxidase (ACO), jasmonate (JA) hydroxylation protein family JASMONATE-INDUCED OXYGENASE (JOX), salicylic acid (SA) degradation protein family Downy Mildew Resistant6 (DMR6) and DMR6-LIKE OXYGENASE (DLO), and strigolactone (SL) biosynthetic protein family LATERAL BRANCHING OXIDOREDUCTASE (LBO). In detail, GA20oxs and GA3oxs catalyze the final two steps of GA biosynthesis: GA20oxs catalyze the conversion of GA_12_ and GA_53_ to GA_9_ and GA_20_, which are converted by GA3oxs to bioactive GA_1_ and GA_4_ [6]. GA2oxs are GA-oxidation enzymes that convert bioactive GAs or their precursors into inactive forms [6]. DAOs catalyze the irreversible conversion of active auxin into inactive 2-oxindole-3-acetic acid (oxIAA) [7]. ACO proteins function in the last step of ethylene biosynthesis by converting ACC into ethylene [8]. JOX proteins hydroxylate jasmonate (JA) into inactive 12-OH-JA [9]. DMR6s, as SA 5-hydroxylases, hydroxylate active salicylate (SA) at the C5 position of the phenyl ring to produce inactive 2,5-DHBA [10]. In *Arabidopsis*, LBO converts methyl carlactonoate into an unidentified strigolactone (SL)-like compound that may be the final product of SL biosynthesis [11]. All of these 2OGD-family hormone biosynthetic and metabolism genes play key roles in maintaining endogenous hormone homeostasis, thereby regulating plant growth and development, and the response to stresses.

2OGDs are non-heme iron-containing proteins. Their catalytic core contains a double-stranded β-helix fold (DSBH) with a highly conserved His-X-Asp-(X)_n_-His (HxD…H) motif, which is responsible for binding Fe (II) to form a catalytic triad [12]. Using Fe (II) as a cofactor and 2-oxoglutarate (2OG) as a co-substrate, 2OGD proteins catalyze oxidation of the substrate and concomitant decarboxylation of 2OG to produce succinate and CO_2_. In addition, a conserved Arg-X-Ser/Thr (RxS/T) motif at the subfamily-conserved position within the secondary structure of the DSBH fold likely binds the C5-carboxy group of 2OG, which is the co-substrate for all known members of the subfamily except isopenicillin N synthase (IPNS), 1-aminocyclopropane-1-carboxylic acid oxidase (ACO) and (S)-2-hydroxypropylphosphonic acid epoxidase (HPPE) [13]. 2OGD-family proteins have been identified in several species [14]. The 2OGD superfamily can be divided into DOXA, DOXB, and DOXC subfamilies based on the amino acid sequence [14]. DOXA proteins contain a 2OG-FeII_Oxy_2 conserved domain, and the DOXA protein AlkB of *Escherichia coli*, which has homologs in *Arabidopsis* and rice, participates in the oxidative demethylation of alkylated nucleic acids and histones [15]. DOXB proteins typically have a conserved 2OG-FeII_Oxy_1 domain; most studies have focused on prolyl-4-hydroxylase, which is involved in the synthesis of cell-wall proteins in plants and algae [16]. DOXC proteins, including those involved in hormone biosynthesis and metabolism, have a conserved 2OG-FeII_Oxy domain [14].

In this study, we identified hormone biosynthesis- and metabolism-related proteins from DOXC family in tomato. Based on analysis of their structures, we predicted their motifs with the aim of determining their molecular mechanisms of action. We also analyzed the transcript levels of these genes in tomato to assess their roles in tomato growth and development, and focused on the correlations between their expression levels and tomato fruit ripening to identify proteins that degrade GAs and auxin during tomato fruit ripening.

## 2. Results

### 2.1. Identification and Phylogenetic Analysis of Hormone Biosynthetic and Metabolism Proteins in 2OGD Superfamily

**Figure 1:**
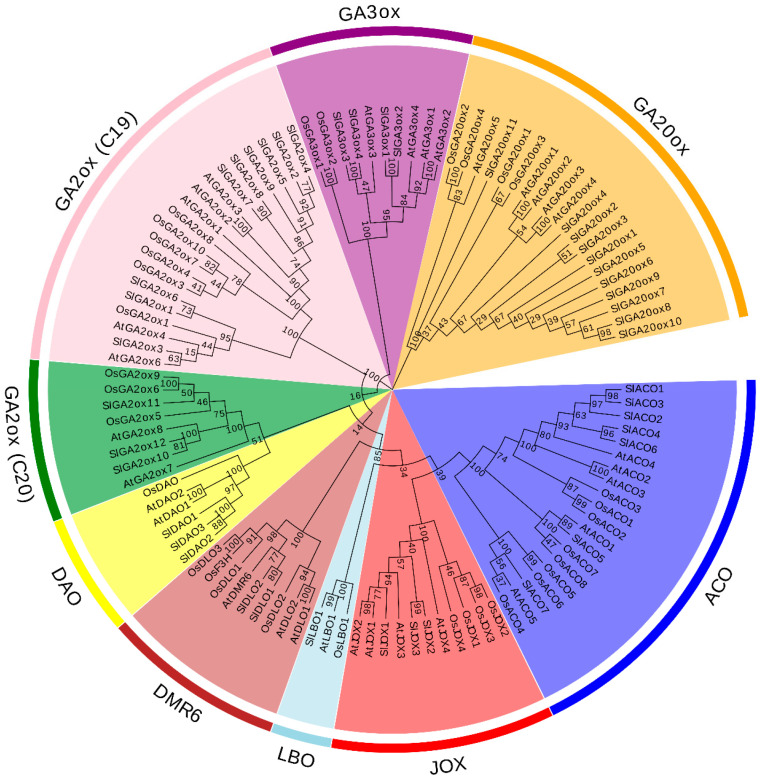
Phylogenetic tree of hormone biosynthetic and metabolism proteins of DOXC family in *Arabidopsis*, rice, and tomato. The phylogenetic tree was constructed by MEGA6 with Maximum likelihood.

Currently known hormone biosynthetic and metabolism proteins in the 2OGD superfamily are exclusively present in the DOXC subfamily. To identify all hormone biosynthetic and metabolism proteins of DOXC family in tomato, we used DOXC-specific 2OGD domain 2OG-FeII_Oxy (PF03171) as a key query in hmmersearch to identify all DOXC proteins in *Arabidopsis*, rice, and tomato. The result showed that 99, 90, and 159 proteins were identified in *Arabidopsis*, rice, and tomato, respectively. A phylogenic tree was constructed using the best-fit model in MEGA6.0, based on the complete sequences of the 348 identified proteins (Figure S1). Nine hormone biosynthetic and metabolism protein families in DOXC family were identified: the GA biosynthetic protein families GA20ox and GA3ox, GA degradation protein families C19-GA2ox and C20-GA2ox, auxin degradation protein family DAO, ethylene biosynthetic protein family ACO, JA hydroxylation protein family JOX, SA degradation protein family DMR, and SL biosynthetic protein family LBO. The bootstrap values were >80%, suggesting high reliability of the results. The numbers of these subfamilies in *Arabidopsis*, rice, and tomato were as follows: 20 GA20oxs, 10 GA3oxs, 20 C19-GA2oxs, 8 C20-GA2oxs, 20 ACOs, 6 DAOs, 11 JOXs, 9 DMR6s, and 3 LBOs. A phylogenetic tree constructed using the above proteins showed that there were 11 GA20oxs, 4 GA3oxs, 9 C19-GA2oxs, 3 C20-GA2oxs, 7 ACOs, 3 DAOs, 3 JOXs, 2 DMR6s, and 1 LBO in tomato, of which 10 GA20oxs (SlGA20ox1-SlGA20ox10), 6 GA2oxs (SlGA2ox2, SlGA2ox4, SlGA2ox5, SlGA2ox7, SlGA2ox8, and SlGA2ox9), 3 DAOs (SlDAO1-SlDAO3), and 5 ACOs (SlACO1-SlACO3, SlACO4, and SlACO6) clustered together to form a monophyletic group (Figure 1). Therefore, these genes emerged via lineage-specific expansion events in tomato. In addition, the identified hormone biosynthetic and metabolism proteins in tomato comprised 104–380 amino acids, and most of them also containing a DIOX_N domain (Table S1).

### 2.2. Synteny and Duplication Analysis of Hormone Biosynthetic and Metabolism Proteins in 2OGD Superfamily

**Figure 2:**
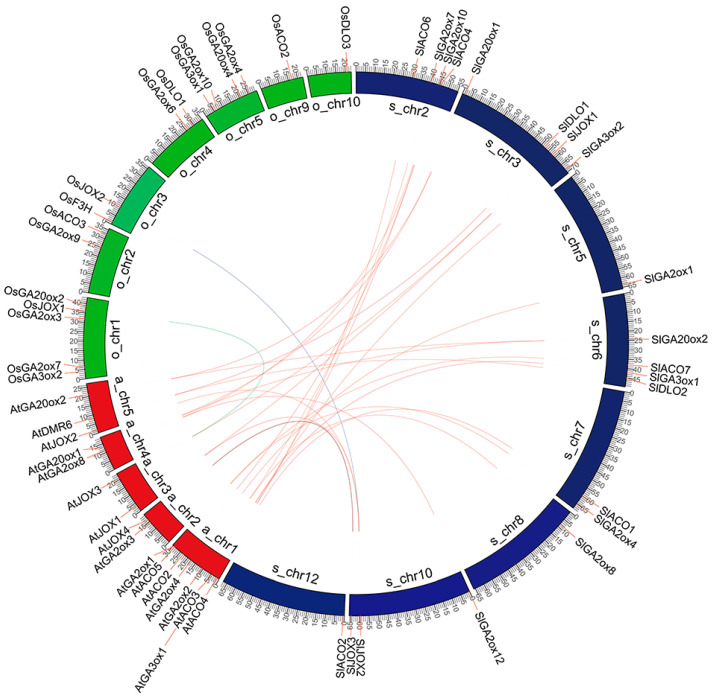
Synteny analysis of hormone biosynthetic and metabolism 2-oxoglutarate-dependent dioxygenase (2OGD) genes among *Arabidopsis*, rice, and tomato. Chromosome numbers of *Arabidopsis* (At), rice (Os), and tomato (Sl) are indicated on the inner side. Red, green, and blue colors represent *Arabidopsis*, rice, and tomato chromosomes. Gene pairs with a collinear relationship are joined by lines. Red lines represent collinear pairs between *Arabidopsis* and tomato, blue lines represent collinear pairs between *Arabidopsis* and rice, green lines represent collinear pairs between rice and tomato.

Synteny was performed to assess the relationships of the hormone biosynthetic and metabolism 2OGD genes among *Arabidopsis*, rice, and tomato. The result showed that there were 27 collinear gene pairs, of which 25 were between tomato and *Arabidopsis*: 5 pairs in the *ACO* family, 2 in the *GA3ox* family, 4 in the *C19-GA2ox* family, 2 in the *C20-GA2ox* family, 6 in the *JOX* family, 4 in the *GA20ox* family, and 2 in the *DMR6* family. There was only one collinear gene pair in the *JOX* family between tomato and rice, as and one between rice and *Arabidopsis* (Figure 2, Table S2). This result is consistent with the evolutionary relationship between monocotyledons and dicotyledons.

The chromosomal location of the hormone biosynthetic and metabolism 2OGD genes in tomato was analyzed based on genome annotation data. The result showed that the identified hormone biosynthetic and metabolism 2OGD genes were unevenly distributed on tomato 12 chromosomes (Figure S2). There was one gene on chromosomes 4, 8, and 12, seven on chromosome 2, and six on chromosome 7. Further, the genes exhibited the following duplication events: nine dispersed gene pairs in *SlGA20ox* (Figure S2, Table S3); two segmental duplication genes (one WGD or segmental duplication events) and two dispersed gene pairs in *SlGA3ox*; seven dispersed gene pairs in *C19-SlGA2ox*; two segmental duplication genes (one WGD or segmental duplication events) in *C20-SlGA2ox*; two tandem duplication events in *SlDAO*; four segmental duplication genes (two WGD or segmental duplication events) in *SlACO*; two segmental duplication genes (one WGD or segmental duplication events) in *SlJOX*; two segmental duplication genes in *SlDMR6* (one WGD or segmental duplication events); and one dispersed gene pair in *SlLBO*.

### 2.3. Multiple Sequence Alignment and Motif Composition Analysis of Hormone Biosynthetic and Metabolism 2OGD Proteins

**Figure 3:**
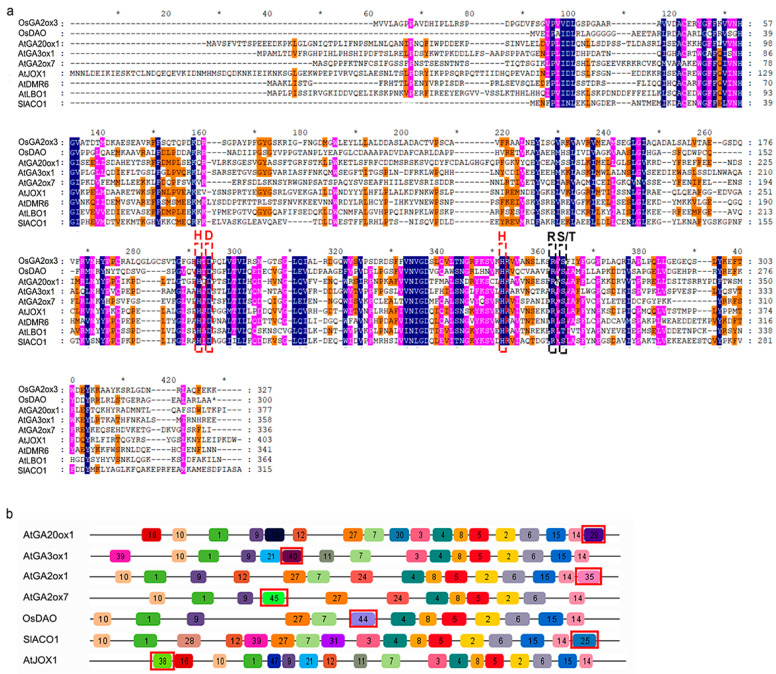
Sequence alignment and conserved motif analysis of functionally characterized hormone biosynthetic and metabolism 2OGD proteins. (**a**) Sequence alignment of functionally characterized hormone biosynthetic and metabolism 2OGD proteins in *Arabidopsis*, rice, tomato. The putative His-X-Asp-(X)_n_-His (HxD…H) and Arg-X-Ser/Thr (RxS/T) motif locations are highlighted in red and black dotted boxes, respectively. (**b**) The motif composition of functionally characterized hormone biosynthetic and metabolism 2OGD proteins. The motif enclosed by red boxes is specific motifs in each group.

To determine the functional similarity of hormone biosynthetic and metabolism 2OGD proteins of tomato with those of *Arabidopsis* and rice, we performed multiple sequence alignments and motif composition analysis. Two 2OGD-family proteins of known three-dimensional structure—OsGA2ox3 and OsDAO [17], and seven hormone biosynthetic and metabolism 2OGD-family proteins—AtGA20ox1, AtGA3ox1, AtGA2ox7, SlACO1, AtJOX1, AtDMR6, and AtLBO1—which have been functionally characterized were aligned to identify conserved domains or motifs in 2OGD family. The result showed that the above 2OGD proteins had the HxD…H and RxS/T conserved motifs in OsGA2ox3 and OsDAO (Figure 3a), which recruit Fe(II) as a cofactor and co-substrate. Further, among the hormone biosynthetic and metabolism 2OGD proteins in *Arabidopsis*, rice, and tomato, SlGA20ox8, SlGA20ox9, SlGA20ox10, SlGA2ox12, and OsACO6 did not have an HxD…H motif, while SlGA20ox7, SlGA20ox10, OsGA2ox10, SlGA2ox12, and OsACO6 lacked an RxS/T motif (Figures S3–S11), suggesting that these proteins do not have 2OGD biological activity.

However, what is the difference of protein structure among different hormone biosynthetic and metabolism 2OGD protein families? Next, we used MEME to identify conserved motifs in DOXC-family proteins of *Arabidopsis*, rice, and tomato (Tables S4 and S5). The result showed that seven hormones biosynthetic and metabolism 2OGD protein families had uniquely conserved motifs—motifs 29, 40, 35, 45, 25, 44, and 38 were unique to the GA20ox, GA3ox, C19-GA2ox, C20-GA2ox, DAO, ACO, and JOX families, respectively (Figure 3b). However, no specific conserved motif was identified in the DMR6 or LBO families (Table S4). Further, sequence alignments showed that SlGA20ox7, SlGA20ox8, SlGA20ox9, and SlGA20ox10 did not have motif 29 (Figure S3), OsGA2ox10 did not have motif 35 (Figure S5), and OsACO4 did not have motif 25 (Figure S7), suggesting that these six proteins are not related to hormone biosynthesis or metabolism. In addition, SlGA2ox6 and SlGA2ox9 were truncated proteins with several missing amino acids in the N-terminal region (Figure S5). In conclusion, from the result of multiple sequence alignment and motif composition, the results suggesting that SlDAO1-SlDAO3, SlGA20ox1-SlGA20ox6, SlGA3ox1-SlGA3ox4, SlGA2ox1-SlGA2ox5, SlGA2ox7-SlGA2ox8, SlGA2ox10-SlGA2ox11, SlACO1-SlACO7, SlJOX1-SlJOX3, SlDLO1-SlDLO2, and SlLBO1 may have the ability of hormone biosynthesis and metabolism in tomato.

### 2.4. Expression of Hormone Biosynthetic and Metabolism 2OGD Genes in Tomato

**Figure 4:**
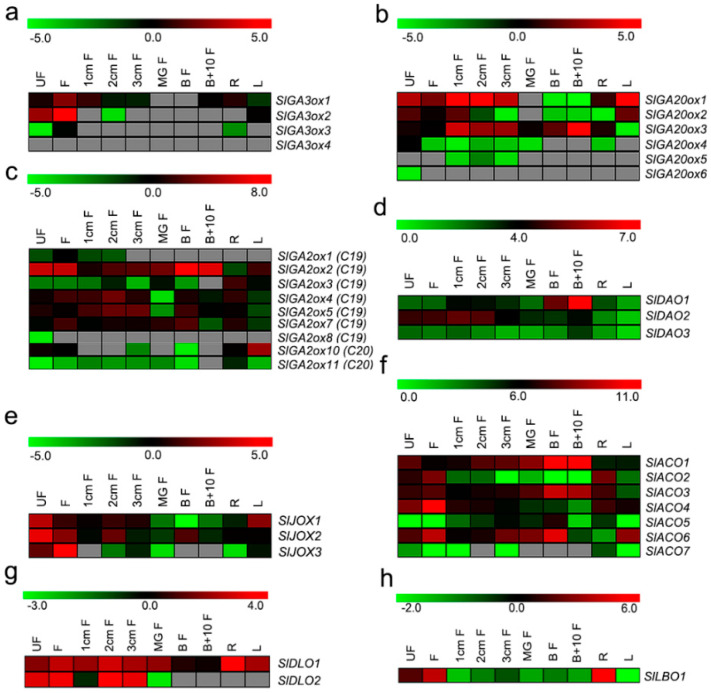
Expression pattern of hormone biosynthetic and metabolism 2OGD genes in tomato. (**a**–**h**) Expression pattern of *SlGA3ox*, *SlGA20ox*, *SlGA2ox*, *SlDAO*, *SlJOX*, *SlACO*, *SlDLO*, and *SlLBO* group genes. Gray boxes represent the expression of genes was undetectable. Unopened flowers (UF); Opened flowers (F); 1 cm fruits (1 cm F); 2 cm fruits (2 cm F); 3 cm fruits (3 cm F); mature-green fruits (Mg F); breaker fruits (Br F); breaker+10 days’ fruits (Br+10 F); roots (R); leaves (L). The detailed descriptions of the stages and tissues were on the website (http://ted.bti.cornell.edu/cgi-bin/TFGD/digital/home.cgi).

To assess the function of identified hormone biosynthetic and metabolism 2OGD genes in tomato, we analyzed online transcriptome data of tomato roots, leaves, flowers, and developing fruits. Most genes exhibited distinct spatial and temporal expression patterns (Figure 4). Three *GA3ox* genes exhibited the highest expression in flowers, *SlGA3ox1* had moderate expression in roots and early developing fruits, and *SlGA3ox2* had moderate expression in leaves. No *GA3ox* gene was expressed during fruit ripening (Figure 4a). Regarding the *GA20ox* family, *SlGA20ox1*, *SlGA20ox2*, and *SlGA20ox3* were highly expressed in flowers and early developing fruits; *SlGA20ox1* and *SlGA20ox3* were also expressed in roots, and *SlGA20ox1* and *SlGA20ox2* were expressed in leaves. *SlGA20ox4* was specifically expressed in unopened flowers. Only *SlGA20ox3* was expressed during fruit ripening, during which its expression increased continuously (Figure 4b). Five GA2ox-family genes (*SlGA2ox3*, *4*, *5*, *7*, and *10*) showed high expression in roots, three (*SlGA2ox2*, *3*, and *10*) in leaves, and six (*SlGA2ox1*, *2*, *4*, *5*, *7*, and *10*) in flowers. In addition, four genes (*SlGA2ox2*, *4*, *5*, and *7*) had high expression in early developing fruits, which increased during fruit ripening (from the mature-green stage to the breaker stage) (Figure 4c). Among the *DAO* family, the expression of *SlDAO1* was high in ripening fruits, moderate in early fruits, and low in roots, leaves, and flowers. *SlDAO2* was expressed mainly in flowers and early fruits, while the expression of *SlDAO3* was negligible in all organs. Notably, *SlDAO1* expression increased significantly from the mature-green to the breaker stage, suggesting a role in fruit ripening (Figure 4d). The expression of the three *JOX*-family genes was highest in flowers, while that of *SlJOX1* and *SlJOX2* was moderate in roots, leaves, and early developing fruits, and *SlJOX2* was expressed in breaker fruits (Figure 4e). Regarding the *ACO* family, three genes (*SlACO2*, *3*, and *4*) were expressed in roots, two (*SlACO4* and *5*) in leaves, and five (*SlACO1*, *2*, *3*, *4*, and *6*) in flowers. Further, four genes (*SlACO1*, *3*, *4*, and *6*) had high expression in early developing fruits, and the expression of four other genes (*SlACO1*, *3*, *5*, and *6*) increased from mature-green to breaker fruit (Figure 4f). The *DLO*-family gene *SlDLO1* showed high expression in roots, leaves, flowers, and early fruits, and decreased expression in ripening fruits, while *SlDLO2* was expressed only in flowers and early fruits (Figure 4g). The only *LBO* gene in tomato, *SlLBO1,* was expressed mainly in roots and flowers, suggesting roles in root and flower development (Figure 4h). In conclusion, a variety of 2OGD hormone biosynthetic and metabolism genes play roles in organ development and fruit ripening in tomato.

### 2.5. Expression of SlGA2ox and SlDAO Genes during Tomato Fruit Ripening

**Figure 5:**
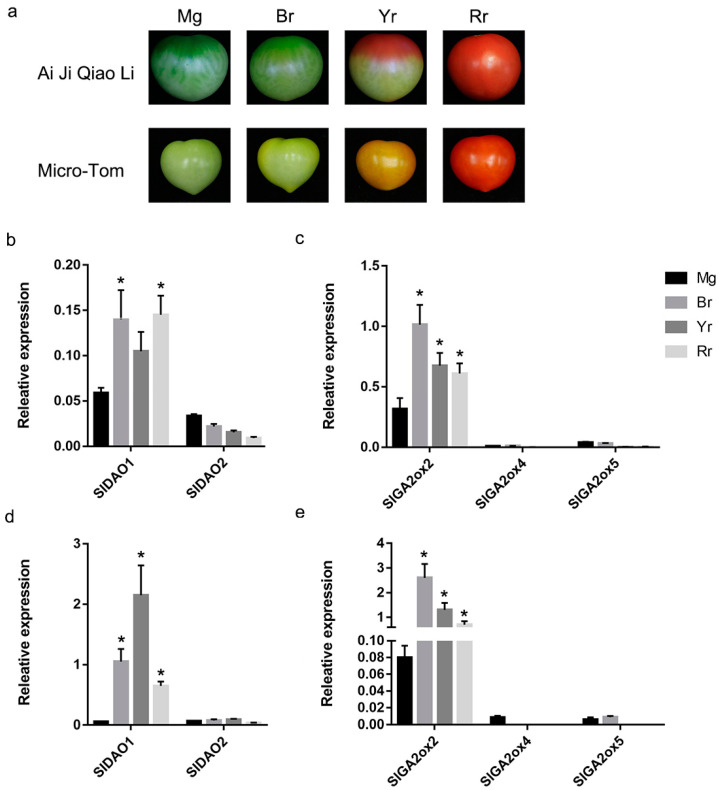
Expression analysis of *SlDAOs* and *SlGA2oxs* genes during tomato fruit ripening in the pericarp. (**a**) Different ripening stages of Ai Ji Qiao Li and Micro-Tom. (**b**) Expression levels of *SlDAOs* in Ai Ji Qiao Li. (**c**) Expression levels of *SlGA2ox* genes in Ai Ji Qi Li. (**d**) Expression levels of *SlDAOs* in Micro-Tom. (**e**) Expression levels of *SlGA2ox* genes in Micro-Tom. Mg: mature-green; Br: breaker; Yr: yellow-ripening; Rr: red-ripening. * The asterisk at the top of each column indicates a significant difference compared to Mg fruits at *p* < 0.05 (*n* = 3) by students t-test.

Ethylene is the major hormone regulating tomato fruit ripening, while auxin and GAs regulate fruit ripening via the ethylene pathway [2,3,4]. The endogenous auxin and GA concentration was decreased during tomato fruit ripening (Figure S12) [3,4], so we investigated the roles of auxin- and GA-degradation genes on tomato fruit ripening. Tomato pericarps at four stages (mature-green, breaker, yellow-ripening, and red-ripening) were collected from the tomato cultivars ‘Ai Ji Qiao Li’ and ‘Micro-Tom’ for qPCR analysis (Figure 5a). The *SlDAO1* expression level was higher than that of *SlDAO2* in Ai Ji Qiao Li and Micro-Tom during fruit ripening (Figure 5b,d). Notably, the expression of *SlDAO1* significantly increased, about two-fold, in Ai Ji Qiao Li, and tenfold in Micro-Tom from the mature-green to the breaker stage; its expression level remained elevated in the yellow- and red-ripening stages. However, *SlDAO2* expression did not significantly change from the mature-green to the breaker stage, and remained very low in the yellow- and red-ripening stages (Figure 5b,d). Thus, *SlDAO1*, rather than *SlDAO2*, likely plays a role in the transition from the mature-green to the breaker stage and subsequent fruit ripening. In addition, the expression of *SlGA2ox2* was 100-fold higher than that of *SlGA2ox4* and *SlGA2ox5*, while *SlGA2ox4* and *SlGA2ox5* expression was negligible in Ai Ji Qiao Li and Micro-Tom (Figure 5c,e). *SlGA2ox2* expression was increased threefold in Ai Ji Qiao Li and thirty-fold in Micro-Tom from the mature-green stage to the breaker stage, and decreased slightly in the yellow- and red-ripening stages (Figure 5c,e); this suggested that *SlGA2ox2* participates in tomato fruit ripening.

### 2.6. Effects of Auxin, GA_3_, and Ethylene on the Expression of SlDAO1, SlDAO2, and SlGA2ox2

**Figure 6:**
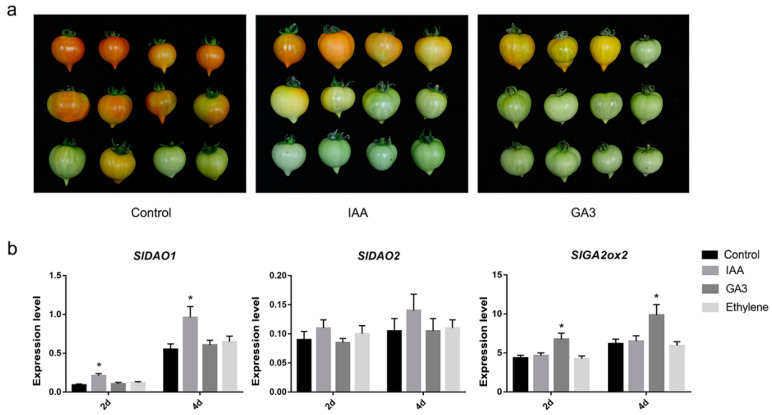
Expression analysis of *SlDAO1*, *SlDAO2*, and *SlGA2ox2* after auxin, GA_3_, and ethylene treatments. (**a**) Photos of mature-green fruits after indole-3-acetic acid (IAA) and gibberellin 3 (GA_3_) treatment, respectively. (**b**) Expression analysis of *SlDAO1, SlDAO2*, and *SlGA2ox2* after auxin, GA_3_, and ethylene treatments. * The asterisk at the top of each column indicates a significant difference at *p* < 0.05 (*n* = 3) by students t-test.

To study the response of *SlDAO1*, *SlDAO2*, and *SlGA2ox2* to auxin, GAs, and ethylene, we treated Micro-Tom mature-green fruits with IAA, GA_3_, and ethylene, and analyzed their expression after 2 and 4 days. Consistent with previous reports, IAA and GA_3_ delayed tomato fruit ripening (Figure 6a). Further, the expression of *SlDAO1* was significantly induced by IAA, but was unaffected by GA_3_ and ethylene at 2 and 4 days, while *SlDAO2* expression was not significantly affected in auxin-, GA-, or ethylene-treated mature-green fruits (Figure 6b). In addition, *SlGA2ox2* showed higher expression in GA_3_-treated fruits, but similar expression in IAA- and ethylene-treated fruits, compared to the control (Figure 6b). In conclusion, the expression of *SlDAO1* and *SlGA2ox2* was induced by auxin and GAs, respectively, suggesting that *SlDAO1* and *SlGA2ox2* are responsible for regulating auxin and GA catabolism during tomato fruit ripening.

## 3. Discussion

### 3.1. Identification of Hormone Biosynthetic and Metabolism Genes from 2OGD Family

The 2OGD superfamily is widespread in microorganisms, fungi, mammals, and plants. In plants, 2OGD proteins are classified as DOXA, DOXB, and DOXC [14]. DOXA proteins are involved in the oxidative demethylation of alkylated nucleic acids and histones, while DOXB proteins are involved in proline 4-hydroxylation in cell-wall protein synthesis, and DOXC proteins in the metabolism of various phytochemicals, such as phytohormones and flavonoids. The number of 2OGDs of the DOXA and DOXB classes is constant across plant species, whereas that of the DOXC class is extremely variable, suggesting that the latter has diversified during the evolution of land plants. The vast majority of 2OGDs from land plants are of the DOXC class, including all hormone biosynthesis- and metabolism-related proteins of the 2OGD family. In this study, the number and classifications of DOXC hormone biosynthesis- and metabolism-related proteins were consistent with the report by Kawal et al. [14]. DOXC proteins are involved in the biosynthesis and metabolism of the phytohormones auxin, GAs, ethylene, JA, SA, and SLs, which play important roles in plant growth and development. Furthermore, the number of DOXC hormone biosynthetic and metabolism genes increases from ancient lower land plants to higher plants, consistent with the high complexity and diversity—and specialized metabolism—of higher plants.

Although the 2OGD superfamily is highly diverse, structural studies suggest that its members have a highly conserved Fe(II) binding HxD/E…H triad motif and a less conserved 2OG C5 carboxy group binding motif (RxS/T) [13]. In this study, forty-three hormone biosynthetic and metabolism proteins of the DOXC family were identified in tomato, but five SlGA20ox7, SlGA20ox8, SlGA20ox9, SlGA20ox10 and SlGA2ox12) lacked the HxD/E…H or RxS/T motif (Figures S3 and S6), suggesting a lack of 2OGD activity. In addition, we identified family-specific conserved motifs in DAOs, GA20oxs, GA3oxs, C19-GA2oxs, C20-GA2oxs, ACOs, and JOXs (Figure 3b); however, their function was unclear. A MdACO1 protein with mutated conserved Lys296 and Arg299 residues in the C-terminal helix retained only 15–30% of the activity of the wild-type, possibly because these two residues are important for ACO activity and may be involved in binding bicarbonate, the unique activator of ACOs [18]. Notably, these two amino acids are located in the ACO-specific conserved motif identified in this study (Figure S7). Therefore, the subfamily-specific conserved motifs may play important roles in the functional differentiation of 2OGD subfamilies.

### 3.2. Functional Analysis of Hormone Biosynthetic and Metabolism Genes in 2OGD Family

GAs, ethylene, auxin, JA, SA, and SLs regulate many aspects of plant growth and development, and the response to stresses. Several 2OGD genes involved in hormone biosynthesis and metabolism have been functionally analyzed in *Arabidopsis* and rice, and these genes participate in the development of roots, stems, flowers, fruits, and seeds. In tomato, the *SlGA20oxs* GA-biosynthetic genes, particularly *SlGA3oxs*, which function in the final step of GA biosynthesis, were mainly expressed in tomato roots, leaves, flowers, and early developing fruits, suggesting that GAs play a role in the development of these tissues/organs (Figure 4a,b). Consistently, RNAi-mediated silencing of *SlGA20ox1*, *SlGA20ox2*, or *SlGA20ox3* affected the development of tomato stems, leaves, fruit, and seeds [19], and inhibitors of GA biosynthesis decreased tomato fruit growth and fruit set; also, exogenous GA_3_ induced parthenocarpic fruits [20,21]. The *SlGA2oxs* GA-metabolism proteins also play key roles in regulating endogenous GA levels. The silencing of *SlGA2ox1-SlGA2ox5* increased the active GA_4_ content, induced parthenocarpic fruits, and inhibited lateral branching in tomato plants [22]. In this study, the newly identified genes *SlGA2ox7* and *SlGA2ox10*, mainly expressed in roots, leaves, flowers, and early developing fruits (Figure 4c), had the same conserved motif as *SlGA2ox1* to *SlGA2ox5* (Figure S5), suggesting a role for *SlGA2ox7* and *SlGA2ox10* in the metabolism of GAs during the development of these tissues/organs.

Although auxin regulates the growth and development of various plant tissues and organs, studies of auxin in tomato have focused on fruit set and development. Exogenous auxin treatment could induce parthenocarpic fruits, and altering the expression of auxin response genes also affected tomato fruit set and development [21,23]. *DAO*-family proteins irreversibly degrade auxin, and a *dao* mutant in rice displayed defective pollen fertility and seed development [7]; meanwhile, a *dao1* mutant in *Arabidopsis* displayed larger cotyledons, increased lateral root density, and elongated pistils [24]. *DAO* has three homologs in tomato; the expression of *SlDAO2* was higher in flowers and early developing fruits compared to *SlDAO1* and *SlDAO3*, suggesting a role in regulating the auxin level for fruit set and development (Figure 4d). Ethylene plays important roles in fruit set and development [25], especially fruit ripening, likely due to high expression of the ethylene-biosynthetic genes *SlACO1*, *SlACO3*, and *SlACO6* in flower, early developing fruits, and ripening fruits (Figure 4f). Other *ACO* genes (*SlACO2* and *SlACO4*) may contribute to ethylene production for root and flower development. In addition, three JA-metabolism *SlJOX* genes showed high expression in tomato flowers (Figure 4e), indicating roles in regulating JA homeostasis for flowering [26]. *AtDMR6,* the product of which degrades salicylic acid, was involved in plant growth and resistance to pathogens, and the *dmr6* mutant displayed smaller size, early senescence, and a loss of susceptibility to *Pseudomonas syringae* pv tomato DC3000 [10]. In tomato, the homolog *SlDLO1* was highly expressed in roots, leaves, flowers, and fruits (Figure 4g), and CRISPR-Cas9 mediated the mutagenesis of *SlDLO1* in tomato conferred broad-spectrum disease resistance; however, vegetative growth and development were not significantly affected, and its role in reproductive organs was not investigated [27]. *SlDLO2* is highly expressed only in flowers and fruits, suggesting roles in regulating the SA level in reproductive organs. SLs are plant hormones that regulate plant root and branch development, as well as stress tolerance [28,29]. High expression of SL biosynthetic and signaling genes in tomato or strawberry fruit indicated roles in fruit development [30]. *LBO* acts in the final stages of SL biosynthesis to produce active SLs in *Arabidopsis*, and its homolog *SlLBO1* is only expressed in roots and flowers (Figure 4h). This suggests that SLs are synthesized in tomato roots and flowers, but does not mean that SLs have no effect on fruit development; they could be transported to fruit from other organs or tissues.

### 3.3. SlGA2ox2 and SlDAO1 May Play a Role in GA and Auxin Metabolism for Normal Ripening of Tomato Fruits

Tomato is a model plant for studying the ripening of climacteric fruits, and ethylene regulates tomato fruit ripening. In this study, exogenous GA_3_ treatment of tomato fruits at the mature-green stage delayed fruit ripening, while overexpression of the GA catabolism gene *SlGA2ox1* specifically in tomato fruits led to early ripening [4]. We have previously shown that GAs play negative roles in the ethylene pathway by inhibiting the expression of ethylene biosynthetic genes (*SlACS2*, *SlACS4,* and *SlACO1*) and signaling genes (*SlETRs* and *SlEINs*) [4]. Therefore, the concentration of GAs in fruits influences fruit ripening in tomato. In plants, the GA level is regulated by the balance between biosynthesis and metabolism. GA20oxs and GA3oxs catalyze the rate-limiting step of active GA biosynthesis, and GA2oxs converts bioactive GAs or their immediate precursors into inactive forms. In this study, although the expression of one *GA20ox* gene (*SlGA20ox3*) increased from the mature-green to the breaker stage (Figure 4b), no *GA3ox* genes, which encode enzymes that catalyze the last step of GA biosynthesis, were expressed (Figure 4a), suggesting the absence of GA biosynthesis in mature-green and breaker fruits. Further, the expression of three GA-metabolism genes (*SlGA2ox2*, *SlGA2ox4*, and *SlGA2ox5*) was increased, and that of *SlGA2ox2* was highest, and dramatically increased, from the mature-green to the breaker stage (Figure 4c). It has been reported that the concentrations of endogenous active GAs (GA_1_ and GA_4_) in the fruit pericarp of tomato decrease significantly from the mature -green to the breaker stage (Figure S12) [4]. Therefore, we speculate that *SlGA2ox2* may be vital for GA metabolism from the mature-green to the breaker stage, and the reduced GA level caused by the increase in *SlGA2ox2* expression promotes tomato fruit ripening.

Auxin also negatively regulates tomato fruit ripening. Exogenous applications of IAA reduced expression of ethylene biosynthetic and consequently reduced ethylene production, and also the ethylene signaling genes, resulting in delayed tomato fruit ripening [1,2]. The concentration of endogenous auxin in tomato fruit pericarps is reduced from the mature-green to the breaker stage (Figure S12) [3]. In plants, auxin is synthesized by tryptophan (Trp)-dependent and -independent pathways [31]. Our knowledge of the genes and intermediates of the Trp-independent pathway is limited, but the complete Trp-dependent pathway has been established. YUCCA (YUC) family proteins function in the final step of Trp-dependent auxin biosynthesis, and play a crucial role in auxin biosynthesis in various plant species. In tomato, six *YUC* genes were identified, the transcript levels of five of which were negligible, whereas one *YUC* gene (*ToFZY4*) displayed high expression during ripening of tomato fruit [32]. It is not clear why the auxin concentration was decreased, but the expression of a key gene in auxin biosynthesis was increased in ripening tomato fruit. One explanation for this is that there is a change from the Trp-dependent to the Trp-independent pathway for auxin biosynthesis between the mature and red-ripe stages of tomato fruits [33], and *ToFZY4* may have a novel function related to tomato fruit ripening rather than auxin biosynthesis. Auxin can be deactivated by conjugation to amino acids, or by chemical oxidation. Conjugation of IAA to amino acids is catalyzed by *GH3*-family proteins and yields, for instance, indole-3-acetic acid aspartic acid (IAA-Asp) and indole-3-acetic acid glutamic acid (IAA-Glu). The chemical oxidation of auxin is catalyzed by DAO-family proteins to produce oxIAA. In tomato, 24 *GH3* genes were identified, only 4 (*SlGH3-1*, *SlGH3-2*, *SlGH3-5*, and *SlGH3-24*) of which showed high expression during fruit ripening [3]. Silencing of *SlGH3-2* in tomato increased the auxin level and reduced lycopene accumulation in ripening fruit, suggesting that *SlGH3-2* plays a role in deactivating free auxin to maintaining normal ripening of tomato fruit [3]. However, oxIAA is a major IAA catabolite, where up to 10–100 folds more oxIAA than the major IAA conjugates IAA-Glu and IAA-Asp was detected in *Arabidopsis* [34,35]. More importantly, oxIAA oxidized by DAO is biologically inactive, and is formed rapidly and irreversibly in plant tissues [34,35,36]. *DAO* is likely involved in maintaining the basal level of active auxin under normal growth conditions, while *GH3* functions in the response to various environmental factors [37]. In this study, we identified three DAO genes in tomato. *SlDAO3* had lost some sequences in the N-terminal (Figure S8), suggesting that it may be not involved in IAA degradation. *SlDAO2* expression was negligible, but that of *SlDAO1* was high and increased from mature-green to breaker fruits (Figure 4d); moreover, it was significantly induced by auxin in mature-green fruits (Figure 6b). These results implicate *SlDAO1*, rather than *SlDAO2* and *SlDAO3*, in auxin metabolism from the mature-green to the breaker stage during tomato ripening. In addition, the reduction in auxin level caused by the increase in *SlDAO1* expression may play an important role in maintaining normal ripening of tomato fruit.

## 4. Materials and Methods

### 4.1. Identification and Phylogenetic Analysis of Hormone Biosynthesis and Metabolism Related DOXC Proteins

To find proteins belonging to DOXC family, we used 2OG-FeII_Oxy (PF03171) domain as query in hmmsearch BLAST of *Arabidopsis*, rice, and tomato protein databases downloaded from JGI [38]. All sequences (length ≥ 100 aa) with an E-value cutoff 1 × 10^−4^ were retrieved. The obtained sequences were submitted to Pfam [39] and SMART [40] to verify the existence of 2OG-FeII_Oxy domain. In order to better understand the relationship among all members of the DOXC and identify proteins involved in hormone biosynthesis and metabolism, we then used all verified protein sequences to construct a phylogenetic tree by MEGA6 with Maximum likelihood. The best model JTT + F was selected by Model Generator software. According to hormone biosynthesis and metabolism related genes with known function in *Arabidopsis* and rice, all proteins which clustered into hormone biosynthesis and metabolism related protein subfamilies were selected to construct a new phylogenetic tree.

### 4.2. Chromosomal Location and Synteny Analysis

Genome annotation files were downloaded from the *Arabidopsis*, rice, and tomato databases to obtain chromosomal location information of these hormone biosynthetic and metabolism genes, then the Circos software was used to draw location pictures. A method similar to that developed for the Plant Genome Duplication Database (PGDD) [41] was used to identify syntenic blocks in *Arabidopsis*, rice, and tomato. Potential homologous sequences were initially identified by BLASTP (E-value < 1 × 10^−5^, top 5 matches). MCScanX was used for synteny analysis [42]. Additionally, MCScanX was further used to detect duplicate types of these biosynthetic and metabolism genes in tomato.

### 4.3. Multiple Sequence Alignment and Motif Composition Analysis

To detect the HxD/E…H and RxS/T motifs, multiple sequence alignments were performed by submitting protein sequences to ClustalW with the default parameters in BioEdit software. Motif composition analysis was performed by submitting protein sequences to MEME [43] with the following parameters: the maximum number of motifs was 50 and the maximum motif length was 15 amino acids.

### 4.4. Expression Analysis

Transcriptome datasets of different tomato organs were downloaded from Tomato Functional Genomics Database [44]. RPKM values of related genes were transformed in log_2_ level, and a heatmap was shown using MeV4.8 software (Dana-Farber Cancer Institute, Boston, MA, USA).

### 4.5. Plant Materials and Hormone Treatments

Two tomato cultivars Ai Ji Qiao Li grown in greenhouse and Micro-Tom grown in climate chamber were chosen as plant materials. The fruit was collected at four different ripening stages: mature-green (Mg), breaker (Br), yellow-ripening (Yr), and red-ripening (Rr). The fruit pericarp sample without placenta and seeds was collected and then immediately frozen in liquid nitrogen prior to storage at −80 °C until RNA extraction.

Tomato cultivars Micro-Tom grown in climate chamber was used for hormone treatments of fruits. Flowers were tagged at the date of pollination. After 36 days, mature-green fruits on the plants were injected with 0.1 mM IAA, 0.1 mM GA_3_, and 0.1 mM ethephon, respectively, distilled water was used as the control. The amount of injection was about 50 µL per fruit. Twelve fruits for each treatment were performed. The fruit pericarp without placenta and seeds was collected at two days and four days after treatments, and were immediately frozen in liquid nitrogen, and then stored at −80 °C. Plant growth conditions was: 16-h light (25 °C)/8-h dark (18 °C) photoperiod cycle and 65% relative humidity. In addition, detached mature-green fruits were injected with 0.1 mM IAA and 0.1 mM GA_3_, respectively, distilled water was used as the control. Then the fruit was placed under dark at 25 °C and 90% relative humidity, photos were taken after eight days.

### 4.6. RNA Extraction and qPCR Analysis of Selected Genes

Total RNA was extracted with a modified CTAB method [4]. cDNA library was generated by Primerscript RT reagent Kit with gDNA Erase (Takara, Beijing, China) according to the manufacturer’s protocol. qPCR was carried out using SYBR Premix Ex Taq II (Takara, Beijing, China). Primer sequences were listed in Table S6. Three biologicals with triplicates were performed and results were analyzed using the 2^−ΔCT^ method. *Actin* gene (gene ID: Solyc11g005330) was used as the reference.

## 5. Conclusions

We have identified 43 hormone biosynthetic and metabolism genes of nine subfamilies of the 2OGD family, which were related to GAs, ethylene, auxin, JA, SA, and SLs in tomato. The subfamily-specific conserved motifs identified in this study might play roles in the functional differentiation of 2OGD subfamilies, and the different expression profiles suggest that these genes play diverse roles in tomato organ growth and development. Especially, the expression levels of the auxin-degradation gene *SlDAO1* and the GA-degradation gene *SlGA2ox2* were significantly increased from the mature-green to the breaker stage during tomato fruit ripening, accompanied by decreased endogenous IAA and GAs levels. In addition, the expression of *SlDAO1* and *SlGA2ox2* was increased by IAA and GA_3_, respectively, indicating that *SlDAO1* and *SlGA2ox2* may be responsible for reducing IAA and GA concentrations to maintain normal ripening of tomato fruit.

## Supplementary Materials

Supplementary materials can be found at https://www.mdpi.com/1422-0067/21/15/5344/s1, Figure S1: Phylogenetic tree of DOXC family proteins identified in *Arabidopsis*, rice, and tomato, Figure S2: Chromosomal location and duplication analysis of hormone biosynthetic and metabolism genes in tomato, Figure S3: Sequence alignment of GA20ox group proteins, Figure S4: Sequence alignment of GA3ox group proteins, Figure S5: Sequence alignment of GA2ox (C19) group proteins, Figure S6: Sequence alignment of GA2ox (C20) group proteins, Figure S7: Sequence alignment of ACO group proteins, Figure S8: Sequence alignment of DAO group proteins, Figure S9: Sequence alignment of Jox group proteins, Figure S10: Sequence alignment of DMR6 group proteins, Figure S11: Sequence alignment of LBO group proteins, Figure S12: The concentrations of GA_1_, GA_4_, and IAA decrease from the mature-green (Mg) to the breaker (Br) stage, Table S1: Identification and characterization of hormone biosynthetic and metabolism proteins in *Arabidopsis*, rice, and tomato, Table S2: Microsynteny relationships of hormone biosynthetic and metabolism genes in *Arabidopsis*, rice, and tomato, Table S3: Duplication modes of hormone biosynthetic and metabolism genes in tomato, Table S4: Motif composition in DOXC class of 2OGD family in *Arabidopsis*, rice, and tomato, Table S5: Motif sequence identified by MEME tools in *Arabidopsis*, rice, and tomato DOXC class of 2OGDs, Table S6: Primers used in this study.

## Abbreviations

GAs	Gibberellins
JA	Jasmonate
SA	Salicylic acid
SL	Strigolactone
GA20ox	GA20-oxidase
GA3ox	GA3-oxidase
GA2ox	GA2-oxidase
DAO	Dioxygenase for Auxin Oxidation
ACO	1-aminocyclopropane-1-carboxylic acid oxidase
JOX	JASMONATE-INDUCED OXYGENASE
DMR6	Downy Mildew Resistant6
DLO	DMR6-LIKE OXYGENASE
LBO	LATERAL BRANCHING OXIDOREDUCTASE
FPKM	Fragments Per Kilobase Per Million
