# Supplementary material for: Identification and Expression Analysis of Hormone Biosynthetic and Metabolism Genes in the 2OGD Family for Identifying Genes That May Be Involved in Tomato Fruit Ripening
Source: Int J Mol Sci. 2020 Jul 28;21(15):5344. doi: 10.3390/ijms21155344 (PMC7432023; doi:10.3390/ijms21155344)
Supplement: Supplementary file 1 [file ijms-21-05344-s001.zip › Supplementary Figures.pdf]

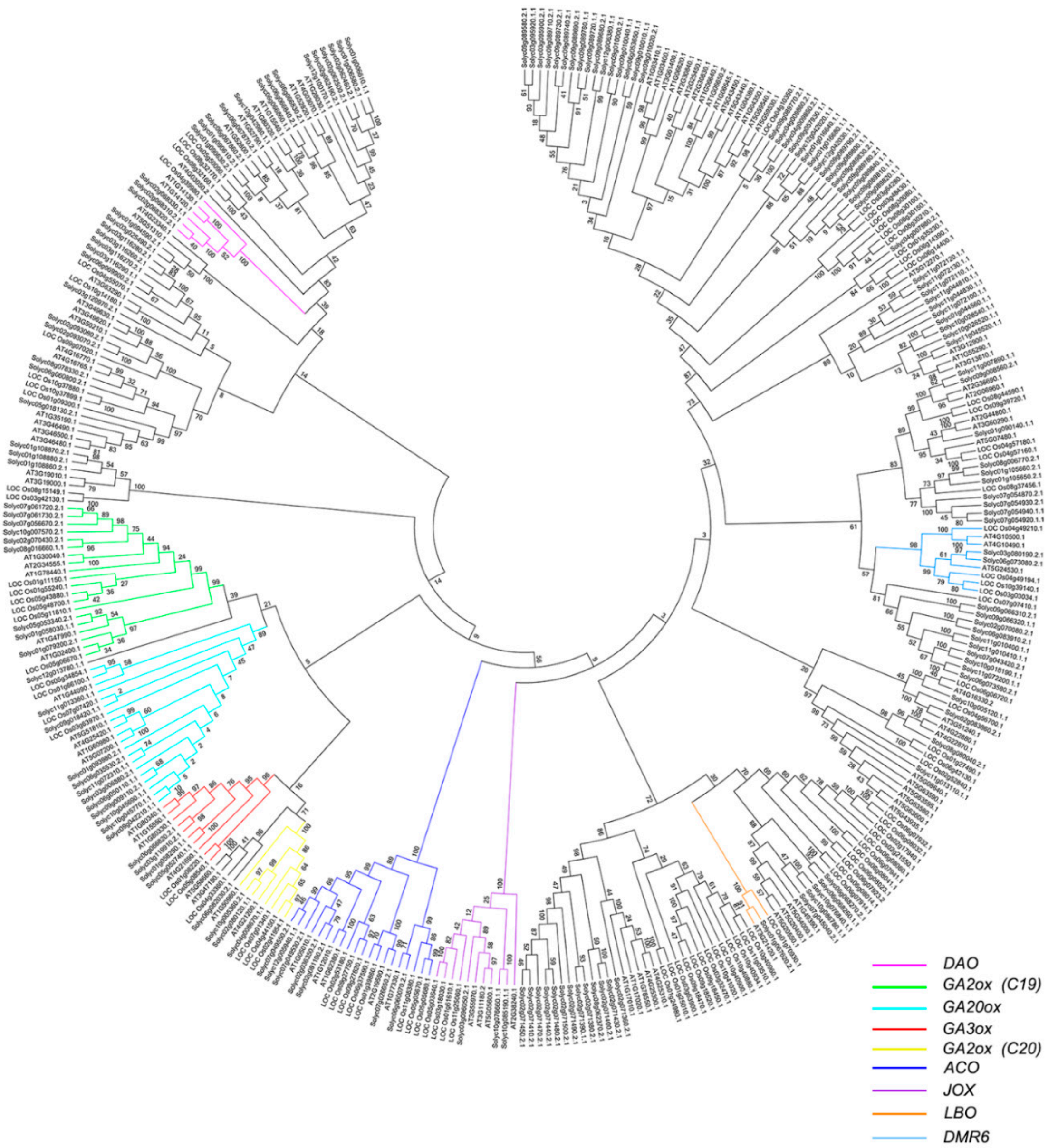

Fig S1. Phylogenetic tree of DOXC family proteins identified in *Arabidopsis*, rice and tomato. Different color lines represent the nine hormones biosynthetic and metabolism protein families.

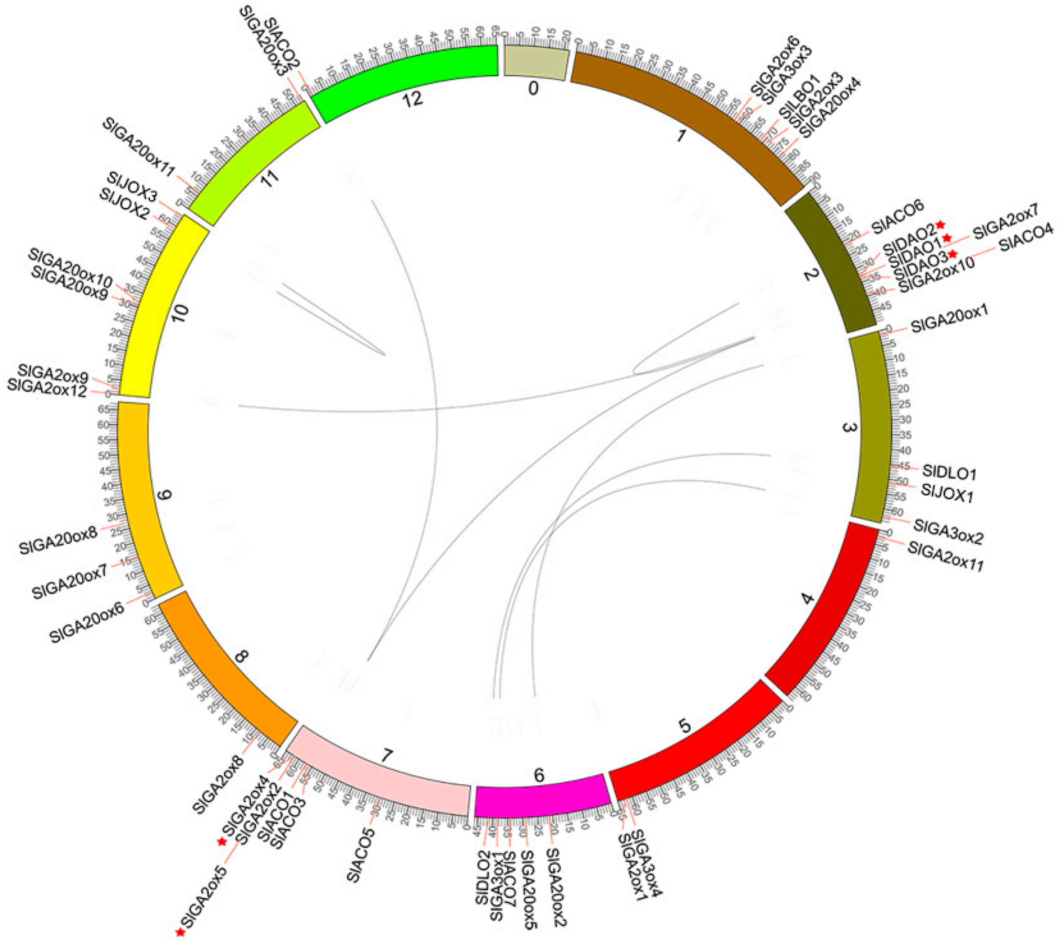

Fig S2. Chromosomal location and duplication analysis of hormone biosynthetic and metabolism genes in tomato. Chromosome number is indicated on the inner side. WGD or segmental duplication gene pairs are joined by black lines. Tandem duplication gene pairs are marked by red stars.

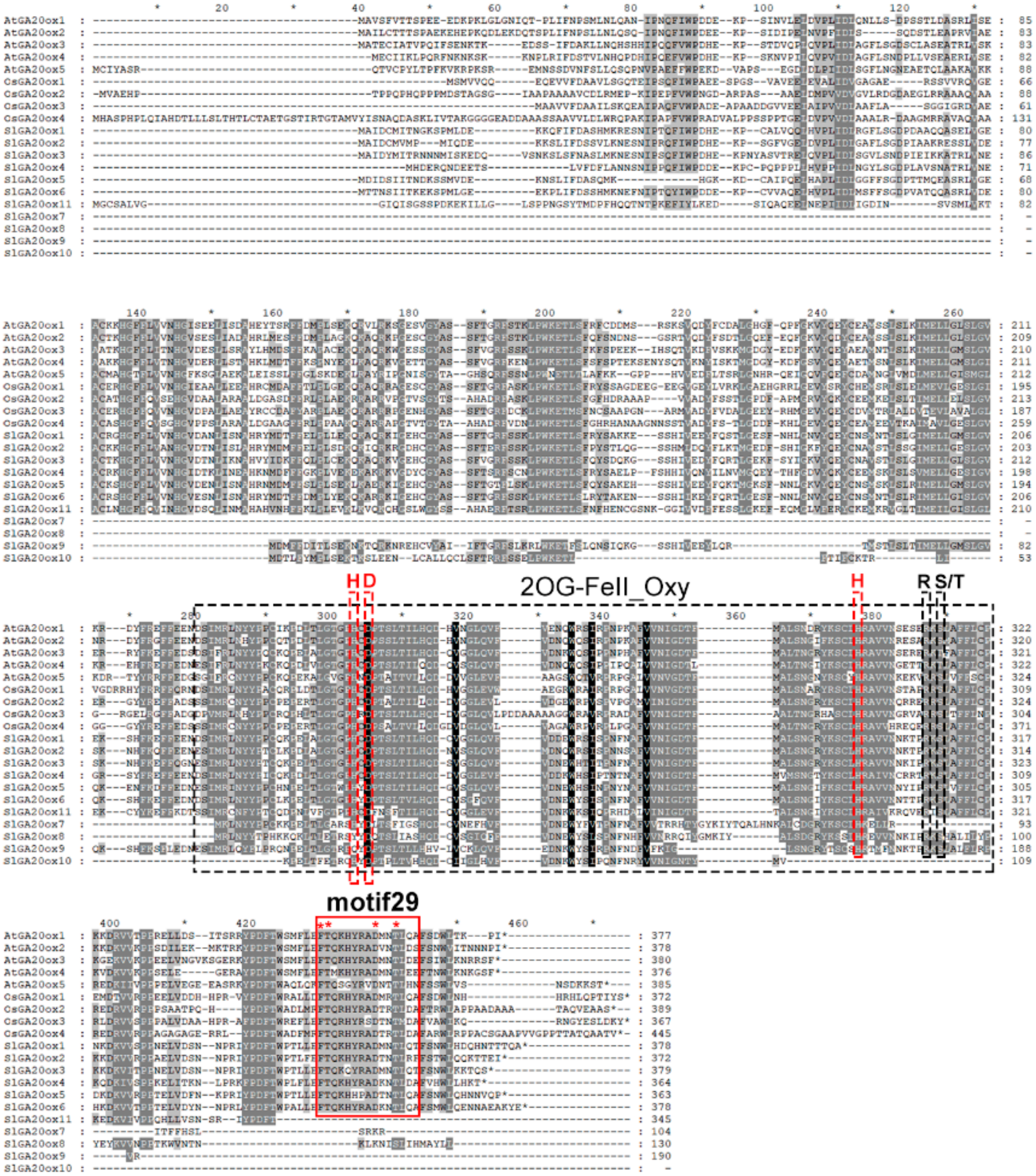

Fig S3. Sequence alignment of GA20ox group proteins. The putative HxD ...H and RxS/T motif locations were highlighted in red and black dotted boxes. The specific motif was enclosed by a red box and the conserved aa sequence was marked by a red asterisk. Bigger black dotted box indicating 2OG-FeII_Oxy conserved domain in each protein.

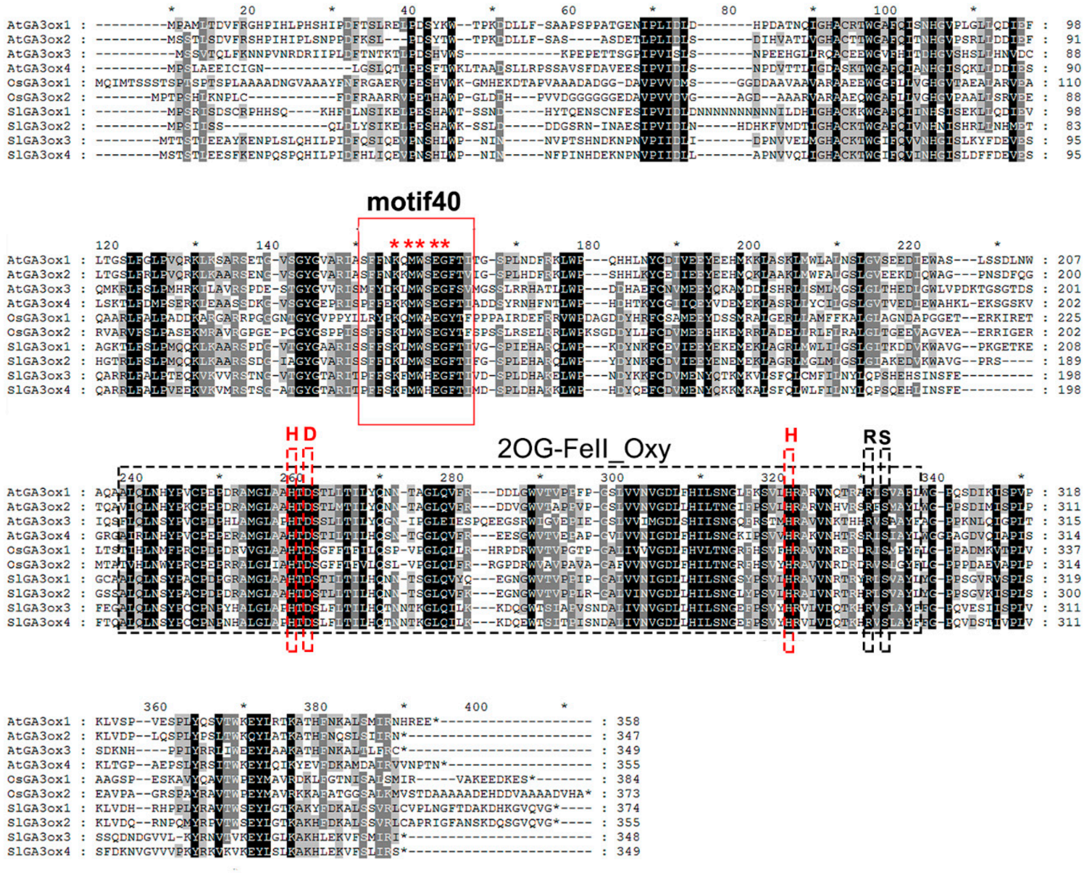

Fig S4. Sequence alignment of GA3ox group proteins. The annotation for the figure is the same as above.

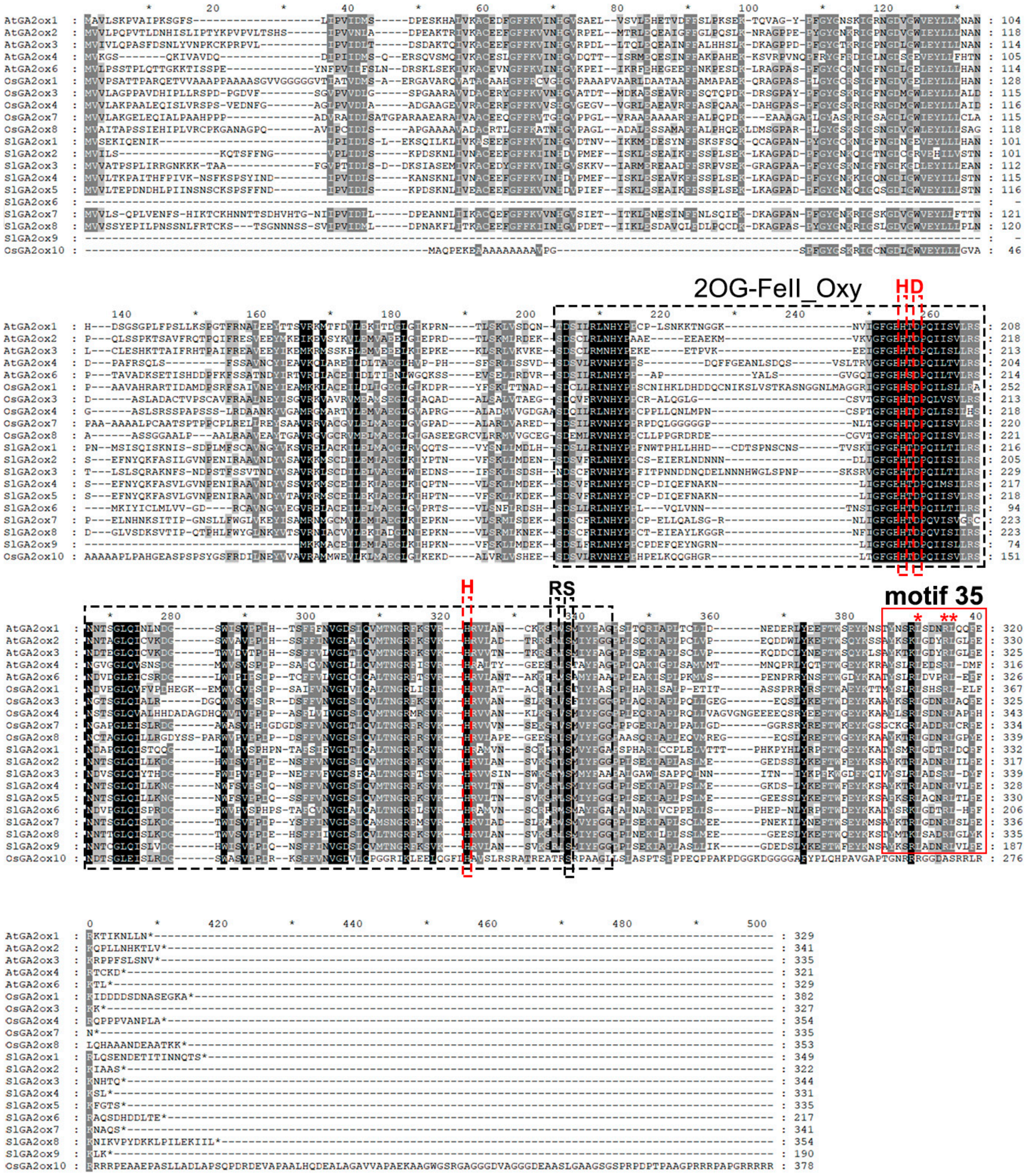

Fig S5. Sequence alignment of GA2ox (C19) group proteins. The annotation for the figure is the same as above.

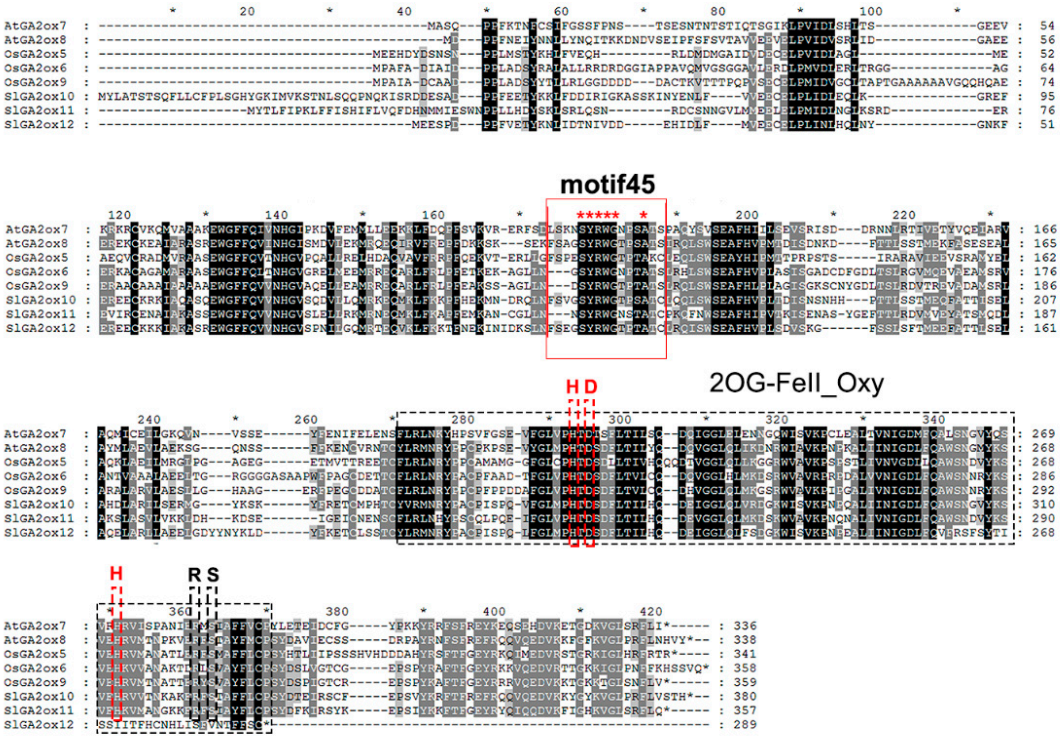

Fig S6. Sequence alignment of GA2ox (C20) group proteins. The annotation for the figure is the same as above.

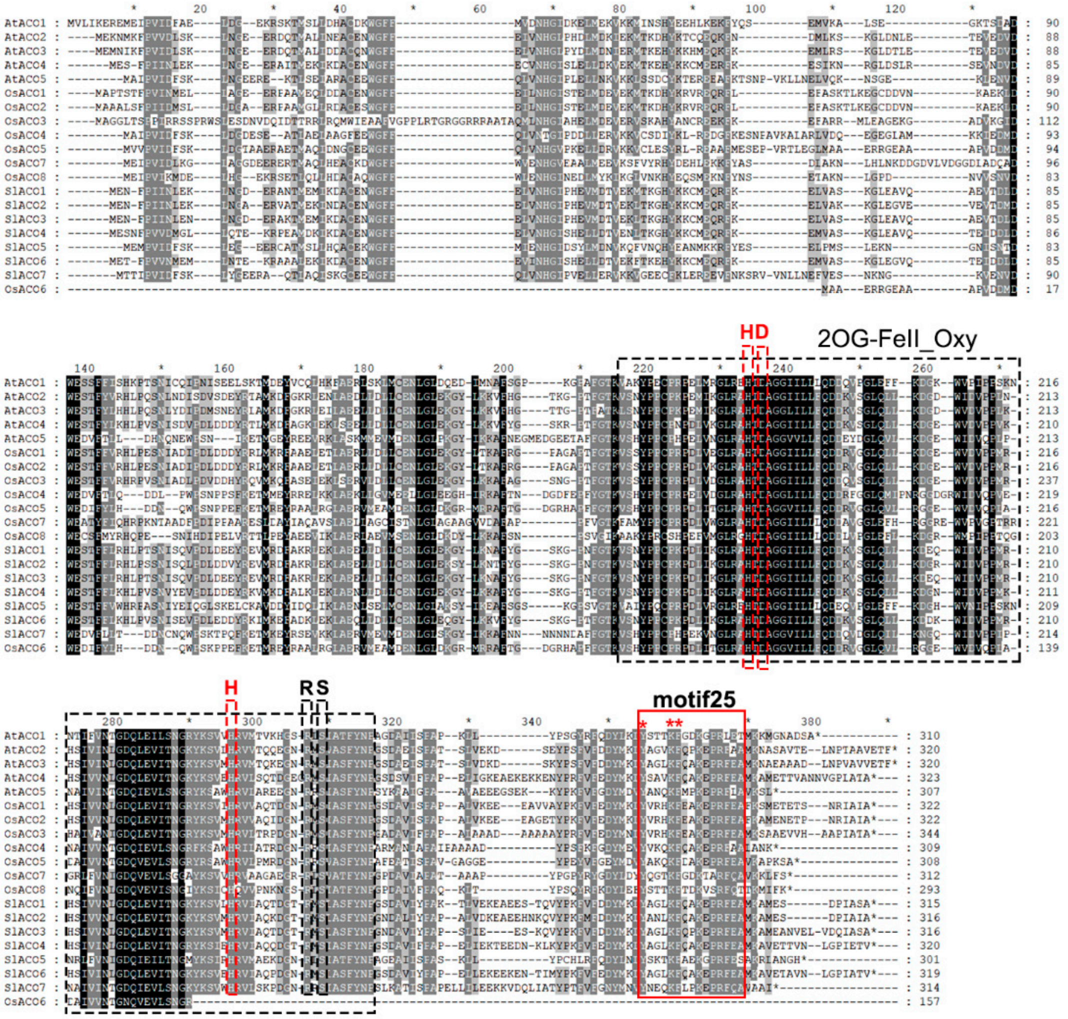

Fig S7. Sequence alignment of ACO group proteins. The annotation for the figure is the same as above.

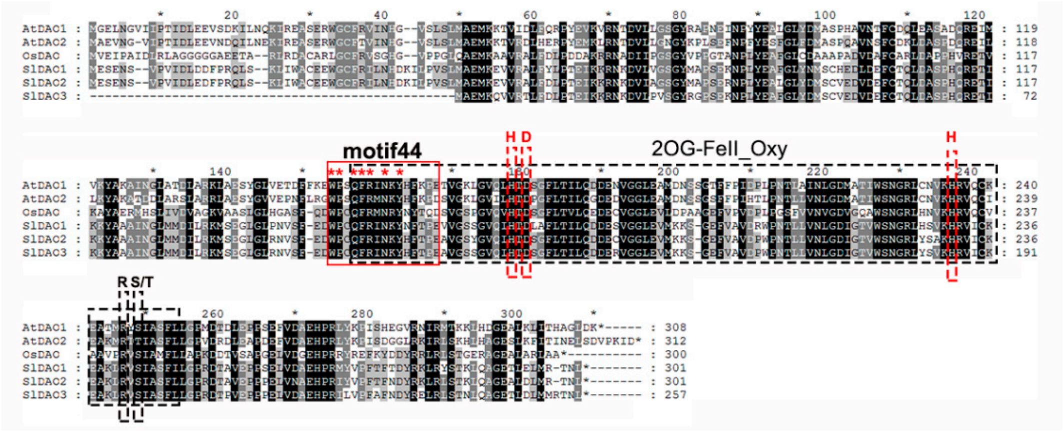

Fig S8. Sequence alignment of DAO group proteins. The annotation for the figure is the same as above.

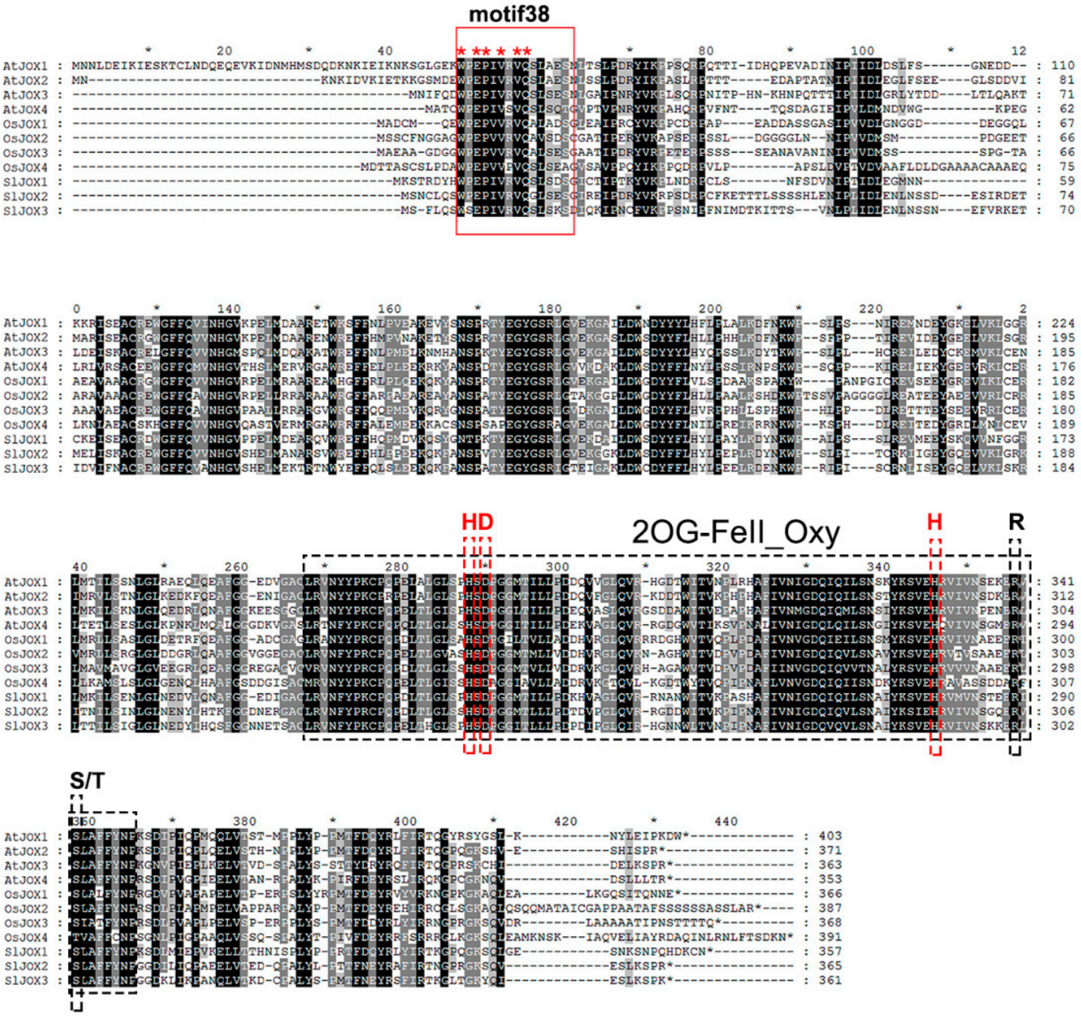

Fig S9. Sequence alignment of Jox group proteins. The annotation for the figure is the same as above.

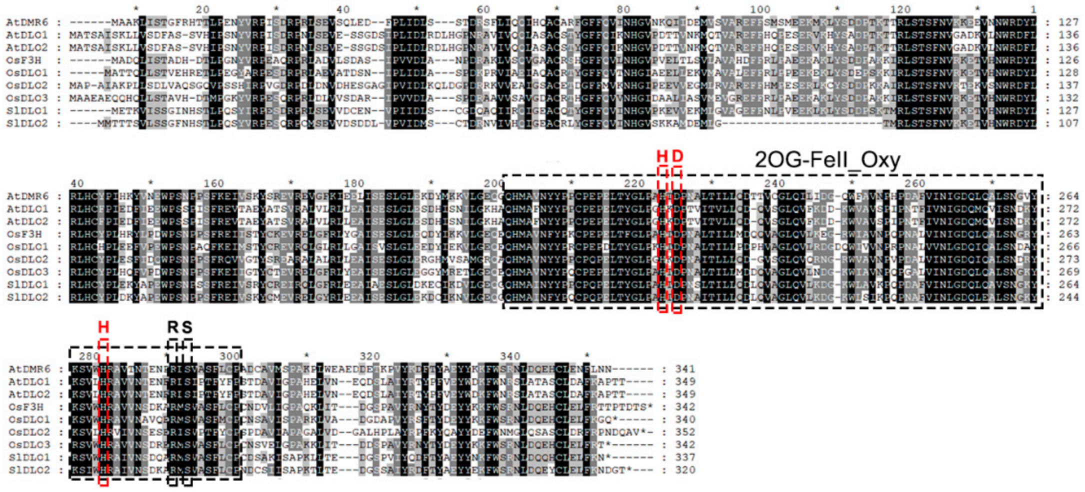

Fig S10. Sequence alignment of DMR6 group proteins. The putative HxD ...H and RxS/T motif location were highlighted in red and black dotted box. Bigger black dotted box indicating 2OG-FeII_Oxy conserved domain in each protein.

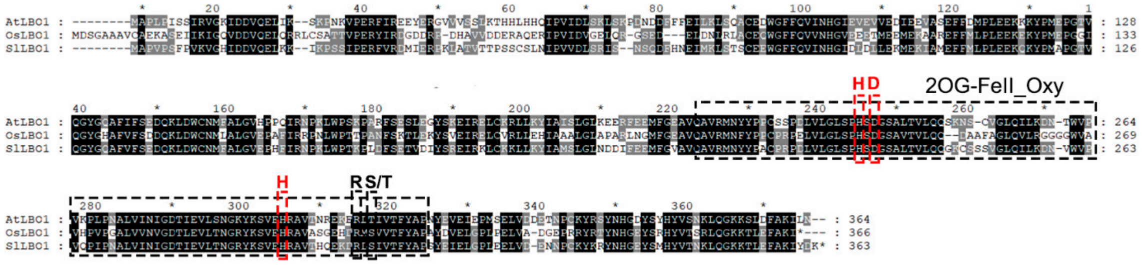

Fig S11. Sequence alignment of LBO group proteins. The putative HxD ...H and RxS/T motif location were highlighted in red and black dotted box. Bigger black dotted box indicating 2OG-FeII_Oxy conserved domain in each protein.

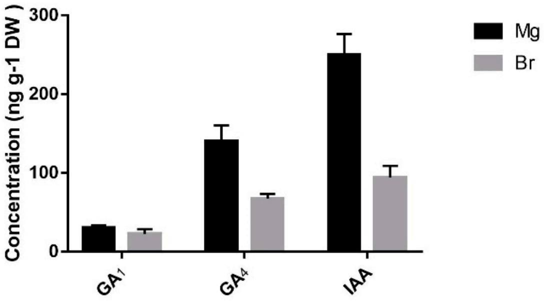

Fig S12. The concentrations of GA_1, GA_4, and IAA decrease from the Mature-green (Mg) to the Breaker (Br) stage. The concentrations of GA_1 and GA_4 are from our published data (reference 4).
